# *Caravan-Qual:* A global scale integration of stream water quality observations into a large-sample hydrology dataset

**DOI:** 10.1038/s41597-026-07352-7

**Published:** 2026-05-02

**Authors:** Edward R. Jones, Frederik Kratzert, Michelle T. H. van Vliet

**Affiliations:** 1 Department of Physical Geography, Faculty of Geosciences, Utrecht University, Utrecht, The Netherlands; 2 Google Research, Vienna, Austria

## Abstract

Protecting and improving surface water quality is contingent upon understanding the trends and spatial patterns in physical, biological, and chemical conditions and their underlying drivers. This requires observational data, spanning a diverse range of water quality constituents, coupled with contextual environmental data. Here we present the first global-scale integration of stream water quality into large-sample hydrology (named *Caravan-Qual*), combining ~96 million observations of 100 constituents with streamflow measurements, meteorological forcing and catchment attributes covering the period 1980–2025. We envisage that the dataset can facilitate a diverse range of empirical analyses (e.g. spatio-temporal analysis across diverse regions, quantification of pollutant loadings and exports, concentration-discharge analysis), in addition to supporting development and evaluation of process-based and data-driven models for water quality prediction and management.

## Background & Summary

Hydrological monitoring is critical for the sustainable management of Earth’s water resources, with data underpinning our understanding of natural processes in water systems and the impact of anthropogenic interventions. Furthermore, observational data is fundamental for the development, calibration and validation of process-based models, remote sensing and data driven approaches (e.g. machine learning) that both further our process understanding and support water resource management^1^.

The last decade has seen a proliferation in efforts to compile, standardise and openly disseminate datasets spanning hundreds to thousands of catchments, driven by the emergence of *large-sample hydrology* (LSH) as a sub-discipline in hydrological sciences^2,3^. Many of these datasets have emerged following the CAMELS (Catchment Attributes and MEteorology for Large-sample Studies) framework first developed for the contiguous United States^4^, with national implementations publicly available for Australia^5^, Brazil^6^, Chile^7^, Denmark^8^, France^9^, Germany^10^, Great Britain^11^, India^12^, Spain^13^ and Switzerland^14^. Other noteworthy LSH datasets include the Hydrometeorological Sandbox - École de Technologie Supérieure (HYSETS)^15^ and LArge-SaMple Data for Hydrology and Environmenal Sciences (LamaH) datasets for Europe^16^, Central Europe^17^ and Iceland^18^.

Leveraging these national efforts, the *Caravan* initiative^19^ has sought to harmonise and combine multiple datasets into a single, globally-applicable framework. Together with publicly available extensions (e.g. GRDC-Caravan^20^), *Caravan* currently contains accessible, consistently formatted and globally standardised daily streamflow observations, together with associated meteorological forcing (e.g. precipitation, temperature)^21^ and static attributes (e.g. land use, soils)^22^, for ~26,000 catchments distributed globally. Such LSH datasets have already enabled novel applications in hydrological modelling, including the development of machine learning approaches for streamflow prediction^23^.

Comparable advances have not (yet) been made for water quality research, for which the availability and accessibility of observational data lags considerably behind streamflow^24^. While national and regional water quality datasets exist, such as for the US^25^, China^26^ and the European Union^27^, these datasets are typically inconsistent in aspects such as monitored water quality constituents, naming conventions and reporting units. Efforts to aggregate and standardise observational water quality data from these sources into global databases, such as the Global Freshwater Quality Database (GEMStat)^28^, GLObal RIver CHemistry Database (GLORICH)^29^ and Global River Water Quality Archive (GRQA)^30^, provided new opportunities to examine water quality dynamics at regional to global scales. However, these datasets lack connections to catchment attributes and meteorological forcings that are integral to other LSH datasets, while connections to observed streamflow measurements are also often lacking. Given the inextricable link between water quality and streamflow (e.g. dilution, mobilisation and transport), this represents a significant shortcoming of existing global water quality databases^31^. Recently, several national level datasets that include water quality have been developed, including for the USA (CAMELS-Chem)^31^, Switzerland (CAMELS-CH-Chem)^32^ and Germany (QUADICA v2)^33^. Additionally, curated water quality datasets that include some hydrographic information also exist^34,35^. Nevertheless, the overall lack of water quality in large sample hydrology datasets represents a critical bottleneck for advancing our understanding of water quality dynamics at large scales.

Here, we present an open-access dataset (named *Caravan-Qual*) that combines observations of 100 water quality constituents with corresponding streamflow records, catchment attributes and meteorological forcing time series. To this end, we leverage a range of national to global water quality datasets, together with both data and open-source software associated with the *Caravan* initiative.

This work represents the first attempt to bring observational water quality data into large-sample hydrology at global scale, integrating water quality with catchment attributes, meteorological forcing and co-located streamflow observations, and is envisaged to facilitate research into topics including:

- Spatio-temporal analyses of stream water quality dynamics at regional to global scales.
- Investigation of the empirical relationships between (constituent-specific) water quality responses and hydrological, meteorological and catchment characteristics.
- The development and evaluation of process-based, hybrid and data-driven water quality models across diverse hydrological and climatic conditions.

It is also hoped that the open-access nature and standardised format of *Caravan-Qual*, together with an introductory Jupyter notebook, helps lower barriers to entry for researchers interested in water quality, while simultaneously promoting transparency and reproducible science within the field.

## Methods

### Water quality data: sources

**Fig. 1:**
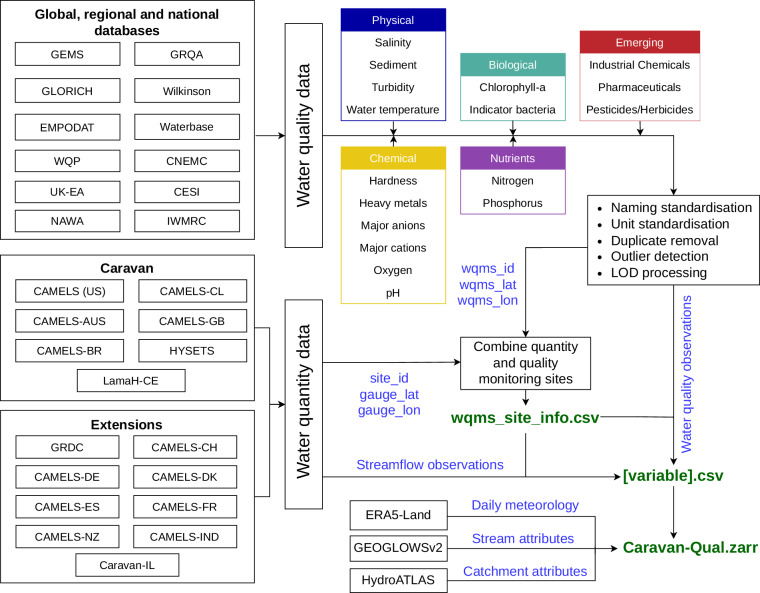
Methodological workflow for creating the *Caravan-Qual* database, highlighting the sources of water quality and streamflow observations and the subsequent processing steps. All code for replicating this workflow is freely and openly available at: https://github.com/SustainableWaterSystems/Caravan-Qual.

Observational water quality data in *Caravan-Qual* is compiled from several existing global, regional and national databases (Fig. 1), all of which are freely available under open licenses that permit redistribution (see the licenses/ folder for full details), including:

- **Global:** UNEP GEMS/Water Global Freshwater Quality Archive (GEMS)^28^
- **Global:** Global River Water Quality Archive (GRQA)^30^
- **Global:** GLObal River Chemistry (GLORICH) dataset^29^
- **Global:** Wilkinson pharmaceutical data^36^
- **Europe:** NORMAN EMPODAT^37^
- **Europe:** Waterbase WISE State of Environment (Waterbase)^27^
- **United States:** Water Quality Portal (WQP)^25^
- **China:** China National Environmental Monitoring Centre (CNEMC)^26^
- **United Kingdom:** Department for Environment, Food and Rural Affairs (UK-EA)^38^
- **Canada:** Canadian Environmental Sustainability Indicators (CESI)^39^
- **Switzerland:** National Surface Water Quality Monitoring Programme (NAWA)^32^
- **Iran:** Iran Water Resources Management Company (IWMRC)^40^

A total of 100 water quality constituents are included, categorised into five groups and 18 sub-groups (Table 1), representing some of the most commonly monitored water quality constituents which are relevant for human activities and environmental health (Table 1). These constituents cover a large diversity in both source (e.g. primarily natural versus anthropogenic) and in-stream behaviour (e.g. conservative versus reactive transport). Note that these groupings serve only as an organisational framework; all individual constituents are included separately in *Caravan-Qual* with a unique constituent name and code (Table 1). The metadata of each water quality dataset was initially screened to identify the presence of and naming conventions used for each of the 100 target water quality constituents in *Caravan-Qual*, with all relevant observations systematically downloaded from each water quality dataset and mapped to a common nomenclature (Table 1).

**Table 1:** Groupings, sub-groupings and constituents included in the *Caravan-Qual* dataset.

Group	Subgroup	Constituent	Constituent code(s)	Units
Physical	Salinity	Electrical Conductivity	EC	μS/cm
		Total Dissolved Solids	TDS	mg/l
	Sediment	Total Suspended Solids	TSS	mg/l
	Turbidity	Turbidity	Turbidity	NTU
	Water temperature	Water Temperature	TEMP	deg C
Chemical	Carbon^1^	Dissolved Carbon	DC; DIC; DOC	mg/l
		Particulate Carbon	PC; PIC; POC	mg/l
		Total Carbon	TC; TIC; TOC	mg/l
	Hardness	Total Hardness	Hardness-Tot	mg/l
	Heavy metals^2^	Arsenic	As-Dis; As-Tot	μg/l
		Cadmium	Cd-Dis; Cd-Tot	μg/l
		Chromium	Cr-Dis; Cr-Tot	μg/l
		Copper	Cu-Dis; Cu-Tot	μg/l
		Iron	Fe-Dis; Fe-Tot	μg/l
		Lead	Pb-Dis; Pb-Tot	μg/l
		Mercury	Hg-Dis; Hg-Tot	μg/l
		Nickel	Ni-Dis; Ni-Tot	μg/l
		Potassium	K-Dis; K-Tot	mg/l
		Zinc	Zn-Dis; Zn-Tot	μg/l
	Major anions^2^	Chloride	Cl-Dis; Cl-Tot	mg/l
		Fluoride	F-Dis; F-Tot	mg/l
		Hydrogencarbonate	HCO3	mg/l
	Major cations^2^	Calcium	Ca-Dis; Ca-Tot	mg/l
		Magnesium	Mg-Dis; Mg-Tot	mg/l
		Manganese	Mn-Dis; Mn-Tot	μg/l
	Oxygen^3^	Biochemical oxygen demand	BOD; BOD5; BOD7	mg/l
		Chemical oxygen demand	COD; CODCr; CODMn	mg/l
		Dissolved oxygen	DO	mg/l
		Dissolved oxygen saturation	DOSAT	%
	pH	pH	pH	—
Biological	Chlorophyll-a	Chlorophyll-a	Chl-a	μg/l
	Indicator bacteria	Escherichia Coli	EColi	cfu/100 ml
		Fecal Coliform	FC	cfu/100 ml
		Total Coliforms	TotColi	cfu/100 ml
Nutrients	Nitrogen^4^	Dissolved nitrogen	DIN; DKN; DON	mg/l
		Particulate nitrogen	PN; PON	mg/l
		Total nitrogen	TAN; TDN; TIN; TKN; TN; TON	mg/l
		Nitrite	NO2N	mg/l
		Nitrate	NO3N	mg/l
		Nitrate-nitrite	NOxN	mg/l
		Ammonia-nitrogen	NH3N	mg/l
		Ammonium nitrogen	NH4N	mg/l
	Phosphorus^5^	Dissolved phosphorus	DIP; DOP	mg/l
		Particulate phosphorus	POP	mg/l
		Total phosphorus	TDP; TIP; TOP; TP; TPP; TRP	mg/l
Emerging	Industrial Chemicals	Benzene	Benzene	μg/l
		PFOA	PFOA	μg/l
		PFOS	PFOS	μg/l
		Trichloroethene	Trichloroethene	μg/l
	Pharmaceuticals and personal care products (PPCPs)	Amoxicillin	Amoxicillin	μg/l
		Atenolol	Atenolol	μg/l
		Caffeine	Caffeine	μg/l
		Carbamazepine	Carbamazepine	μg/l
		Diclofenac	Diclofenac	μg/l
		Ibuprofen	Ibuprofen	μg/l
		Metoprolol	Metoprolol	μg/l
		Naproxen	Naproxen	μg/l
		Propranolol	Propranolol	μg/l
		Sulfamethoxazole	Sulfamethoxazole	μg/l
		Triclosan	Triclosan	μg/l
	Pesticides/ Herbicides	Atrazine	Atrazine	μg/l

Note that where constituents have multiple constituent codes, each code refers to a distinct form of that constituent.

^1^Various forms of carbon are included: DC = dissolved carbon; DIC = dissolved inorganic carbon; DOC = dissolved organic carbon; PC = particulate carbon; PIC = particulate inorganic carbon; POC = particulate organic carbon; TC = total carbon; TIC = total inorganic carbon; TOC = total organic carbon; ^2^The extensions “-Dis” and “-Tot” in constituent codes are used to distinguish between dissolved and total forms, respectively, of chemical constituents (where applicable); ^3^BOD5 and BOD7 refer to the biological oxygen demand after 5 and 7 days, respectively; ^4^Various forms of nitrogen are included: DIN = dissolved inorganic nitrogen; DKN = Dissolved Kjeldahl Nitrogen; DON = Dissolved Organic Nitrogen; PN = Particulate Nitrogen; PON = Particulate Organic Nitrogen; TAN = Total Ammonia Nitrogen; TDN = Total Dissolved Nitrogen; TIN = Total Inorganic Nitrogen; TKN = Total Kjeldahl Nitrogen; TN = Total Nitrogen; TON = Total Organic Nitrogen; ^5^Various forms of phosphorus are included: DIP = Dissolved Inorganic Phosphorus; DOP = Dissolved Organic Phosphorus; POP = Particulate Organic Phosphorus; TDP = Total Dissolved Phosphorus; TIP = Total Inorganic Phosphorus; TOP = Total Organic Phosphorus; TP = Total Phosphorus; TPP = Total Particulate Phosphorus; TRP = Total Reactive Phosphorus.

### Water quality data: processing and harmonisation

Obtaining observational water quality data for many constituents and from multiple sources presents processing and harmonisation challenges, including (aforementioned) naming conventions, but also related to reporting units, outliers and detection limits^30,41^.

Data processing was implemented in two stages. Individual datasets are first pre-processed independently to remove observations without specific geographical (e.g. latitude and longitude) and temporal (e.g. DD/MM/YYYY) information. Measurement units are harmonised to a target unit per water quality constituent (Table 1), following the procedure described for GRQA^30^, while observations with missing or incompatible units were dropped.

All (remaining) observations are subsequently combined into a unified dataset for post-processing. Individual water quality monitoring sites are identified on the basis of unique coordinate pairs (at 5 decimal places), and were assigned a unique id (“wqms_id”). Any exact duplicate observations (i.e. identical wqms_id, date and value) are removed, retaining a single instance of each.

In *Caravan-Qual*, observations reported as below detection limits in their source datasets are flagged (using the notation: “<”). Left-censored observations are processed using Regression on Order Statistics (ROS) for stations meeting data availability criteria (at least 5 detected values; fewer than 50% of the measurements below detection limit). For water quality monitoring stations not meeting these data availability criteria, left-censored observations are substituted with half of the detection limit^42^. In both cases, imputed values are flagged and detection limits are reported to allow users the flexibility to apply alternative censoring strategies or exclude imputed values entirely from their analyses.

Similarly, outliers are detected and flagged (using the notation: “*”). This is based on physically plausible limits defined per water quality constituent (defined in wq_variable_list.csv) and, for water quality monitoring stations meeting a minimum data availability criterion (at least 10 observations), using the interquartile range method (IQR, with a threshold of 5xIQR). This conservative threshold was chosen to flag extreme anomalies but preserve legitimate high values, given that 1) quality control procedures were likely already applied to source datasets; and 2) an additional filtering to physically plausible limits is already applied. Nevertheless, outliers are flagged in *Caravan-Qual* rather than directly removed to preserve the raw observations. As with the left-censored observations, this ensures users retain control over how to handle anomalous values in their analyses.

Overall, the structure of *Caravan-Qual* enables modular updates, for example, the dataset can be extended with additional water quality or streamflow observations without having to reprocess the whole dataset. Technical validation of the procedure for processing and harmonising observational water quality data is provided.

### Streamflow data: sources

Streamflow data in *Caravan-Qual* is compiled by aggregating observations contained in *Caravan*^19^ with various datasets that have subsequently been released (Fig. 1). The open-access datasets that are included are:

**Table:** 

• CAMELS (US)^4^	• CAMELS-CH^14^
• CAMELS-AUS^43^	• CAMELS-CZ^44^
• CAMELS-BR^6^	• CAMELS-DE^10^
• CAMELS-CL^7^	• CAMELS-DK^8^
• CAMELS-GB^11^	• CAMELS-ES^13^
• HYSETS^15^	• CAMELS-FR^9^
• LamaH-CE^17^	• CAMELS-IND^12^
• LamaH-Ice^18^	• CAMELS-NZ^45^
• GRDC-Caravan^20^	• Caravan-IL^46^

Diverging from *Caravan*, streamflow observations are converted to m^3^ s^−1^ (opposed to mm day^−1^) as volumetric discharge is arguably more suitable for water quality applications (e.g. load estimation, concentration-discharge relationships). Nevertheless, catchment areas are included in metadata to allow users to easily convert between units if desired.

### Integrating water quality and streamflow observations

**Fig. 2:**
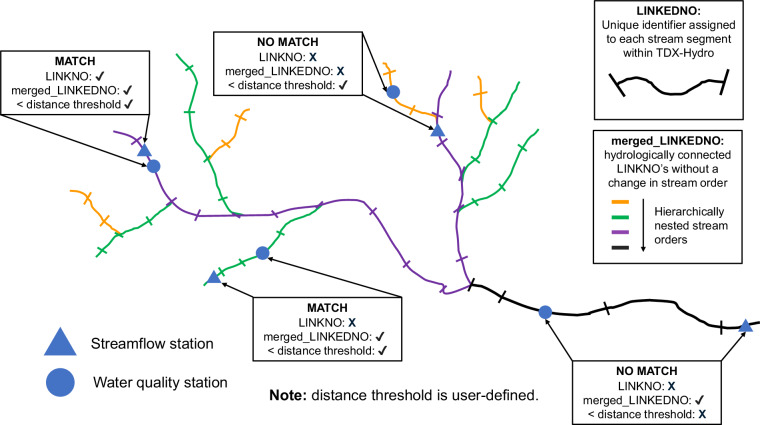
Methodological workflow for matching water quality monitoring stations with streamflow gauges incorporating a user-defined distance threshold.

Water quality monitoring stations are spatially matched to streamflow gauges to integrate water quality and streamflow observations (Fig. 2). To this end, a multi-directional stream network was derived from GEOGLOWSv2^47^ which itself is derived from the TDX-Hydro dataset^48^. We derive an additional variable (named “merged_LINKNO”) from GEOGLOWSv2, which provides a common identifier for connected stream segments (“LINKNO”) that are of the same Strahler stream order (“strmOrder”). Both water quality monitoring stations and streamflow gauges are snapped to the nearest point on this stream network. Water quality monitoring stations are paired with the nearest (upstream or downstream) streamflow gauge on the same “merged_LINKNO”, with along-stream distance included in the metadata to support flexible distance filtering. Technical validation of the approach for matching water quality and streamflow observations is provided.

### Deriving meteorological forcing and catchment attributes

Meteorological forcings and catchment attributes are derived for all water quality monitoring stations using open-source software developed for *Caravan*^19^, which requires lumped upstream catchment polygons per monitoring station. As upstream polygons are not commonly provided in water quality datasets, these were derived using GEOGLOWSv2^47^ by identifying the LINKNO catchment polygon containing each water quality monitoring station and then subsequently aggregating all upstream contributing polygons through recursive network traversal. Additional stream attributes are also derived from GEOGLOWSv2 (Table 2). Technical validation of the delineation of lumped upstream catchments is provided.

**Table 2:** Stream attributes included in *Caravan-Qual*. All variables are processed directly from GEOGLOWSv2, which itself is derived from TDX-Hydro.

Stream attributes	Description
LINKNO	A river ID number unique to the TDXHydro delineation.
strmOrder	The Strahler stream order
DSContArea	The total drainage area upstream of the most downstream point (i.e. the outlet) of this segment.
TDXHydroRegion	The original TDX regional group number
TopologicalOrder	The topological order of a stream
LengthGeodesicMeters	Geodesic length (in meters) of the river segment
TerminalLink	The TDXHydroLinkNo of the eventual outlet
musk_k	The Muskingum k parameter
musk_x	The Muskingum x parameter

Meteorological forcing is derived from the global reanalysis dataset ERA5-Land^21^ for 23 variables (Table 3), while catchment attributes are derived from HydroATLAS^22^ (Table S1). The approaches for deriving these data is extensively described and validated by *Caravan*^19^, with the only deviation being that potential evaporation is estimated from ERA5-Land variables using the FAO’s Penman-Monteith equation (instead of directly using ERA5-Land potential evaporation) due to data quality issues^49^.

**Table 3:** Meteorological variables included in *Caravan-Qual*.

Feature (variable name)	Aggregation	Unit
Air temperature (temperature_2m)	Daily min/max and mean	°C
Dew point temperature (dewpoint_temperature_2m)	Daily min/max and mean	°C
Eastward wind component (u_component_of_wind_10m)	Daily min/max and mean	ms^−1^
Net thermal radiation at the surface (surface_net_thermal_radiation)	Daily min/max and mean	Wm^−2^
Northward wind component (v_component_of_wind_10m)	Daily min/max and mean	ms^−1^
Potential evaporation (potential_evaporation)	Daily sum	mm/day
Precipitation (total_precipitation)	Daily sum	mm/day
Shortwave radiation (surface_net_solar_radiation)	Daily min/max and mean	Wm^−2^
Surface pressure (surface_pressure)	Daily min/max and mean	*kPa*

All variables are processed directly from ERA5-Land using open-source *Caravan* code, with the exception of potential evaporation which is estimated using the FAO’s Penman-Monteith equation.

## Data Records

The full *Caravan-Qual* dataset^50^ is available at 10.24416/UU01-39ZXC4. *Caravan-Qual* is provided in Zarr format, a cloud-optimised format that enables chunked compression, lazy loading and rapid querying of spatial or temporal subsets^51^. The dataset is structured as follows:

- Caravan-Qual.zarr
  - **Dimensions:** time (daily timesteps from 1980-01-01 to 2025-09-30); wqms_id (water quality monitoring station identifier); gauge_id (streamflow gauge identifiers) and LINKNO (river reach identifiers).
  - **Station metadata:** spatial metadata per wqms_id (wqms_lat, wqms_lon, country_name, hydrobasin_level12, merged_LINKNO) and gauge_id (gauge_lat, gauge_lon).
  - **Observational data**: water quality observations, detection limits and flags for multiple parameters (see constituent codes in Table 1), indexed by wqms_id and time. Streamflow observations (streamflow), indexed by gauge_id and time.
  - **Catchment and stream network attributes:** static catchment attributes derived from HydroATLAS^22^ (see Caravan^19^ and Table S1) and stream attributes from GEOGLOWSv2^47^ (see Table 2). Indexed by LINKNO. Catchment attributes are derived as an area-weighted aggregate (see the Aggregation columns in Table S1).
  - **Meteorological data**: daily meteorological forcing from the global reanalysis ERA5-Land dataset (see Table 3), indexed by LINKNO and time.

- Caravan-Qual_linkages.parquet
  - A metadata table (stored in Parquet format) that provides linkages between water quality monitoring stations and their corresponding streamflow gauge identifiers, catchment attributes and meteorological forcing. This file also contains detailed metadata per water quality monitoring station, combined and per individual water quality constituent, including the number of observations, start and end date of monitoring and the number of observation years. This structure enables efficient querying of the entire dataset (based, for example, on geographical, temporal or water quality criteria) without iterating through all data files.
- wqms_site_info.csv
  - Basic metadata for each water quality monitoring station, indexed by station identifier (wqms_id). Contains geographic coordinates (wqms_lat, wqms_lon), the associated river segment identifier (LINKNO), in addition to the co-located streamflow gauge identifier (gauge_id) and distance between the monitoring station and gauge (gauge_distance_km).
- auxiliary/
  - Contains all data required for extending *Caravan-Qual*, such as the GEOGLOWSv2^47^, HydroATLAS^22^ and the raw (unprocessed) water quality data.

For broader accessibility, a lite version of *Caravan-Qual* is available: 10.5281/zenodo.17787065^52^. This includes the water quality observations, in addition to all catchment and streamflow attributes, but linked to monthly (instead of daily) meteorological forcing. Furthermore, all water quality observations are provided here as comma separated values per constituent (i.e. [constituent].csv), including linked streamflow measurements (in m^3^ s^−1^). The “gauge_distance_km” column in “wqms_site_info.csv” allows users to filter linked water quality and streamflow observations by a custom distance threshold suited to their own use case.

## Data Overview

**Fig. 3:**
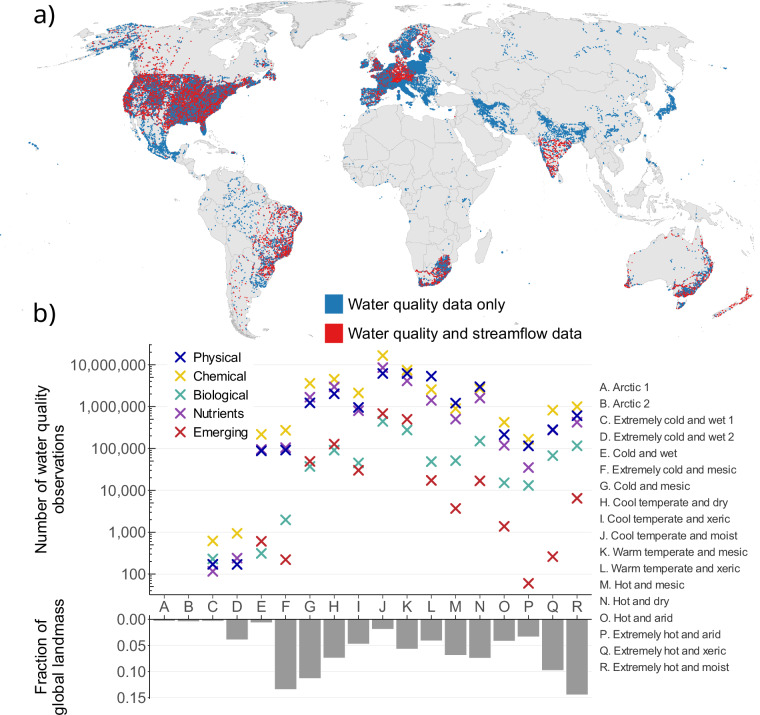
Spatial distribution of stream water quality observations in *Caravan-Qual*. Panel (**a**) displays the location of water quality monitoring stations, including and excluding linkages to a streamflow gauge. Panel (**b**) displays the distribution in the number of water quality observations across five constituent groupings (see Table 1) and the Global Environmental Stratification (GEnS) climate zones^54^. The lower half of panel **b)** shows the fraction of each climate zone on the total land mass.

The current dataset includes >96 million stream water quality observations from 151,814 monitoring stations (across 93,201 unique TDX-Hydro LINKNOs^48^), in addition to >300 million streamflow measurements from 26,207 gauges. *Caravan-Qual* includes observations spanning the time period 1894–2025 which are located across all continents, albeit with a strong spatial bias towards temperate climates of Western Europe (36%), North America (35% of observations) and East Asia & Pacific (12%) (Fig. 3).

**Fig. 4:**
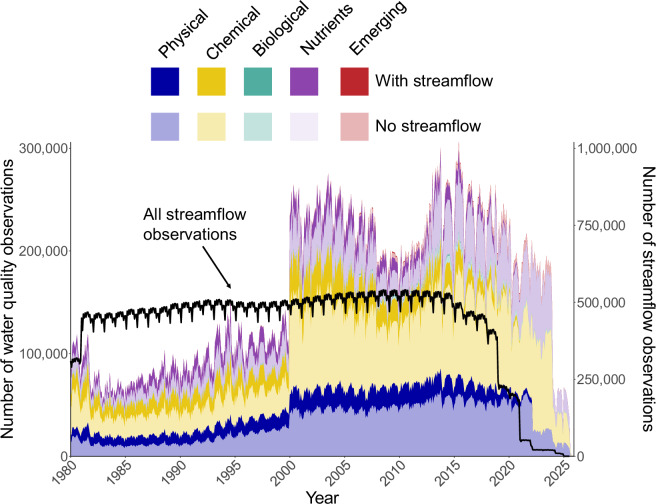
Temporal distribution of water quality observations in *Caravan-Qual* from 1980-2025. Stacked bars (left axis) display the number of water quality observations across five constituent groupings (see Table 1) aggregated per month, with shading distinguishing observations that have (or lack) an associated streamflow observation (with a 10 km distance threshold). The black line (right axis) shows the total number of streamflow observations in *Caravan-Qual*, aggregated per month.

**Fig. 5:**
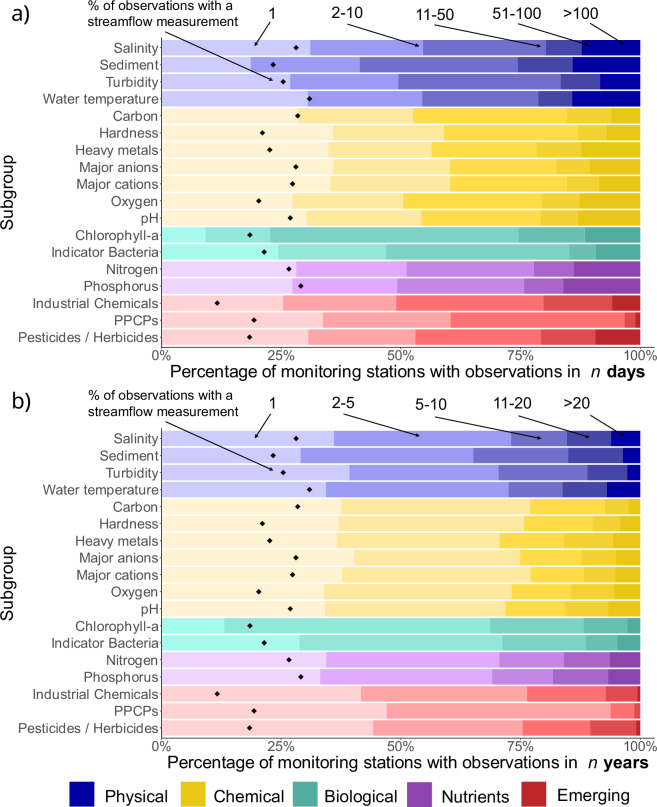
Aggregated statistics on the temporal availability of water quality observations in *Caravan-Qual*. Panel (**a**) displays the percentage of water quality monitoring stations with observations in n **days**, whereas panel (**b**) displays the percentage of stream monitoring stations with observations in n **years**, across 18 constituent subgroupings (see Table 1). Black dots display, per subgroup, the percentage of observations that have an associated streamflow measurement (with a 10 km distance threshold).

The number of observations across all water quality constituents have generally increased over time (the decline in recent years are an artifact of reporting lags), with physical (e.g. water temperature, electrical conductivity) and chemical parameters (e.g. pH, dissolved oxygen) dominating in terms of number of measurements (Figs. 3b, 4). Intra-annual fluctuations are also evident, with a peak in observations between April and August (Fig. 4). This likely reflects increased monitoring during the ecologically sensitive and recreational bathing water seasons of the Northern Hemisphere, which is disproportionately represented in *Caravan-Qual*. Lastly, it should be noted that observational water quality records are highly discontinuous across all constituents, with ~30% of monitoring stations having observations from only a single day, though some longer records do exist for most constituents (Fig. 5).

## Technical Validation

### Processing water quality observations

**Fig. 6:**
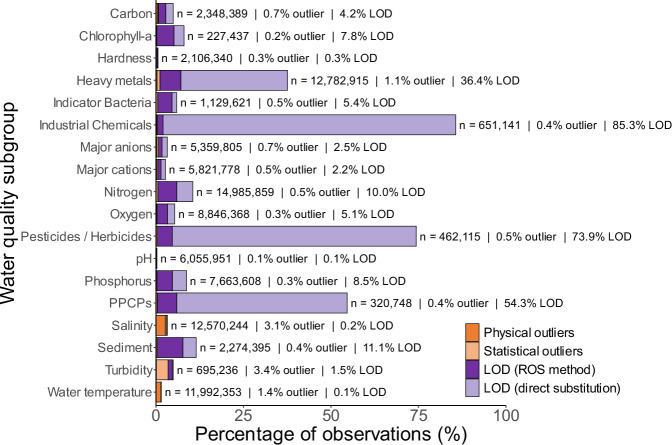
Summary statistics of the water quality observation flagging process in *Caravan-Qual*, showing the percentage of observations flagged as left-censored (processed using Regression on Order Statistics (ROS) or direct Limit of Detection (LOD)/2 substitution) or as outliers (statistical and physical). Note that all raw observations are retained in *Caravan-Qual*, including both and LOD-imputed values, allowing users to apply their own filtering and processing criteria in their analyses.

**Fig. 7:**
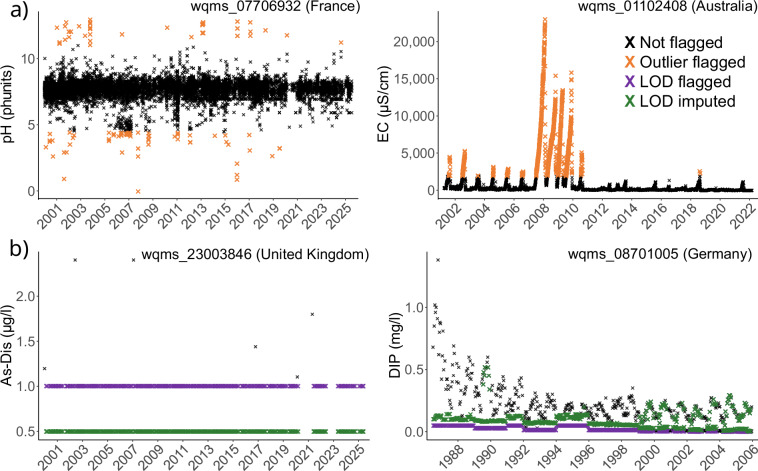
Validation of the water quality processing procedure in *Caravan-Qual*, displaying time-series plots for example stations illustrating (**a**) the flagging of outliers; and (**b**) the flagging and imputation of left-censored observations (i.e. reported with a Limit of Detection [LOD] flag).

Extensive processing was applied to the raw water quality observations that comprise *Caravan-Qual*, including the removal of duplicate observations, outlier detection and procedures for imputing left-censored observational data. To assess the impact of these processing steps on the final dataset, the proportion of observations affected by each processing step is displayed (Fig. 6). Furthermore, outlier detection and LOD imputation are illustrated for example monitoring stations through water quality time series plots (Fig. 7).

Following the removal of exact duplicates across source datasets, *Caravan-Qual* contains ~96 million water quality observations. Across all subgroups, ~1% (1.1 million observations) were flagged as outliers detection (0.5% as physical outliers, 0.4% as statistical outliers) while 10% of observations (11.7 million observations) were flagged as below detection limits. Limit of detection (LOD) processing was highly unequal across water quality subgroupings, being particularly commonplace for industrial chemicals (~85% of observations), pesticides/herbicides (~74%) and PPCPs (~54%), as well as for certain heavy metals (e.g. Mercury, Lead and Chromium). In total, of the left-censored observations, approximately 30% (3.4 million observations) were processed using the ROS method and ~70% (8.2 million observations) using direct substitution of LOD/2.

### Delineation of lumped catchments

To validate the accuracy of the catchment delineation method developed for *Caravan-Qual*, a spatial comparison against catchment boundaries from proximate streamflow gauges is used – given that co-located water quality monitoring stations and streamflow gauges should represent (largely) identical upstream catchment areas.

**Fig. 8:**
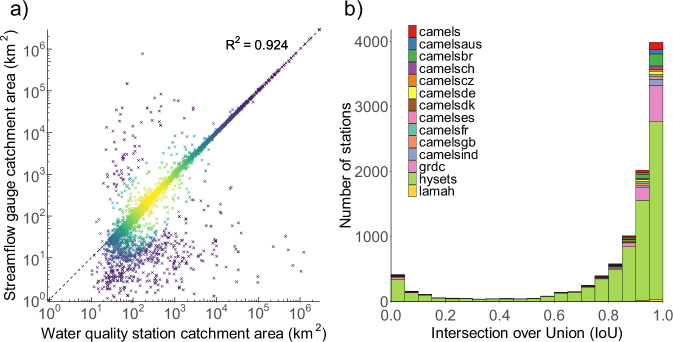
Validation of catchment delineation for water quality monitoring stations using co-located streamflow gauges. Panel (**a**) displays a comparison of catchment areas for water quality monitoring stations (*Caravan-Qual* approach) and streamflow gauges (reported in streamflow datasets) located within 100 m. The dashed line represents 1:1 agreement, with point density displayed using colour gradient. Panel (**b**) displays the distribution of Intersection over Union (IoU) values quantifying spatial overlap between delineated and reported catchment polygons.

9,652 water quality monitoring station-streamflow gauge pairs within a 100 m threshold are evaluated (Fig. 8). Delineated catchment areas for water quality monitoring stations showed strong agreement with reported streamflow gauge areas (R^2^ = 0.92), with a median area ratio (i.e. streamflow gauge area/water quality monitoring station area) of 0.98 (Fig. 8a). Furthermore, the spatial overlap of catchments are high, with a median Intersection over Union (IoU) value of 0.93 (Fig. 8b). 79% of stations (7,589) have a IoU value exceeding 0.8, with 10% (913) having a value less than 0.4. Overall, these results demonstrate that the method developed for *Caravan-Qual* can reliably delineate catchment boundaries for water quality monitoring stations, which are subsequently used for the derivation of catchment characteristics and meteorological forcing.

### Linking water quality (wqms_id) and streamflow (gauge_id) observations

**Fig. 9:**
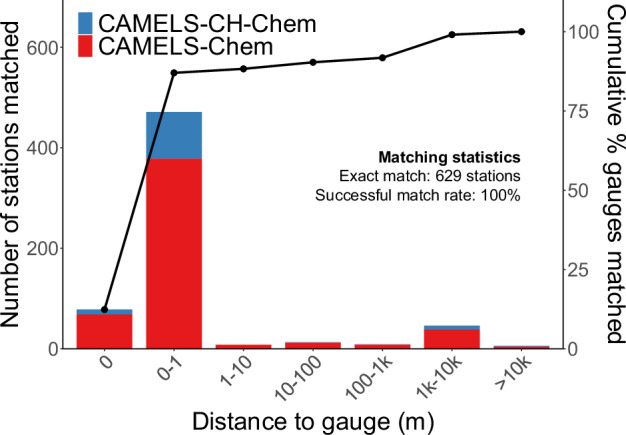
Validation of water quality monitoring station to streamflow gauge matching procedure developed for *Caravan-Qual* against reference datasets (CAMELS-Chem and CAMELS-CH-Chem). Stacked bars display the number of stations matched within successive distance-to-gauge intervals, while the black line shows the cumulative proportion of gauges matched.

To validate the procedure for matching water quality monitoring stations with streamflow gauges in *Caravan-Qual*, we cross-checked our assignments for 631 water quality monitoring stations previously matched in CAMELS applications (516 from *CAMELS-Chem*^14^; 115 from *CAMELS-CH-Chem*^32^) (Fig. 9). Our automated approach achieved a 100% agreement rate with those datasets, successfully identifying the same water quality monitoring – streamflow gauge pairs in all 631 cases. While the vast majority of these water quality monitoring stations are co-located with streamflow gauges (e.g. 549 stations within 1 m distance), water quality monitoring stations were also successfully matched with streamflow gauges at less proximate distances, including 46 water quality monitoring stations located between 1–10 km from the streamflow gauge (Fig. 9). This agreement demonstrates the reliability of our approach for linking water quality monitoring stations and streamflow gauges, and therefore is applicable to the broader *Caravan-Qual* dataset. Nevertheless, we provide users with the flexibility to define their own distance thresholds for matching water quality monitoring stations with streamflow gauges. Table 4 demonstrates how streamflow coverage varies with distance threshold, from 10.2% of stations (17.1% of observations) matched at a distance of 1 km to 34.1% of stations (34.4% of observations) with a more permissive threshold of 50 km. It should be noted that the proportion of matched observations exceeds the proportion of matched stations (Table 4), suggesting that water quality monitoring stations that are matched with a streamflow gauge tend to have longer or more densely sampled observational records.

**Table 4:** Effect of distance threshold on the proportion of water quality monitoring stations and observations matched to streamflow gauges and observations, respectively.

Distance	Water quality monitoring stations with matched streamflow gauges(%)	Water quality observations with matched streamflow observations (%)
<1 km	10.2%	17.1%
<5 km	16.9%	22.5%
<10 km	22.5%	26.2%
<25 km	30.3%	31.3%
<50 km	34.1%	34.4%

## Usage Notes

- *Caravan-Qual* contains stream water quality observations spanning the time period 1894–2025, which are available via the .csv files. The .zarr data stores cover 1980–2025, a period over which >90% of stream water quality observations were collected. Unless otherwise stated, summary statistics presented in this manuscript pertain to the .csv files.
- Significant efforts have been made into retrieving water quality and streamflow observations from across the world, but certain regions (e.g. Sub-Saharan Africa, Middle East and North Africa) are still severely underrepresented. *Caravan-Qual* is designed in such a way to be extendable, either with new streamflow measurements or water quality observations, and it is our hope that the dataset can continue to grow as more observations become available with an open and redistributable license.
- Nevertheless, spatio-temporal biases in *Caravan-Qual* are substantial and users should be aware of their potential implications. Uneven spatial coverage may bias global and regional assessments towards well-monitored regions (e.g. North America, Western Europe), while uneven temporal sampling (both inter- and intra- annual) may bias analyses of seasonal assessments and trend detection.
- To a large degree, the validity of the stream water quality observational data is dependent upon the individual procedures and reporting practices from the monitoring institutes. Water quality observations are subject to multiple sources of uncertainty, including analytical measurement error, transcription and reporting mistakes, and intentional censoring of values.
- Similarly, the catchment attributes and meteorological forcings included in *Caravan-Qual* are subject to uncertainties inherent in the source products (HydroATLAS and ERA5-Land, respectively) and those introduced by spatial aggregations to the catchment level. Nevertheless, both represent state-of-the-art datasets which are compatible with the global scope of *Caravan-Qual*.
- The dataset is accompanied by an interactive Jupyter notebook to provide a user-friendly entry point for researchers seeking to leverage this resource. This can be accessed at: https://github.com/SustainableWaterSystems/Caravan-Qual.

## Supplementary information

Supplementary Information File

## Data Availability

The full *Caravan-Qual* dataset is available at 10.24416/UU01-39ZXC4. For broader accessibility, a lite version of *Caravan-Qual* is available at 10.5281/zenodo.17787065.

## Code availability

The code that was used to produce the *Caravan-Qual* dataset is available through Zenodo^52^ and in a project homepage at: https://github.com/SustainableWaterSystems/Caravan-Qual. The original *Caravan* code is available through Zenodo^53^ and in a project homepage at: https://github.com/kratzert/Caravan/.
